# Recent advances and conceptual changes in the classification of neuroendocrine tumors of the thymus

**DOI:** 10.1007/s00428-021-03037-1

**Published:** 2021-02-08

**Authors:** Hanibal Bohnenberger, Philipp Ströbel

**Affiliations:** Institute of Pathology, University Medical Center Göttingen, Robert-Koch-Str. 40, D-37075 Göttingen, Germany

**Keywords:** Neuroendocrine, Carcinoid, Atypical, Large cell neuroendocrine carcinoma, NET G3, Thymus, Molecular, Classification, World health organization, Sequencing

## Abstract

Neuroendocrine tumors of the thymus (TNET) are exceedingly rare neoplasms. Their histomorphology is identical to neuroendocrine tumors elsewhere in the body (in particular the lungs) and bears no similarity with thymomas and thymic carcinomas. Recent molecular findings have profoundly changed our perception of these tumors and may impact future histological classification systems.

## General features of thymic neuroendocrine tumors

Neuroendocrine tumors of the thymus (TNET) are exceedingly rare neoplasms. Their histomorphology is identical to neuroendocrine tumors elsewhere in the body (in particular the lungs) and bears no similarity with thymomas and thymic carcinomas. Based on the many shared features between pulmonary and thymic NET, TNET and pulmonary NET (PNET) are traditionally classified using the same criteria into typical and atypical carcinoids (TC and AC), large cell neuroendocrine carcinomas (LCNEC), and small cell carcinomas. Although previous studies found genomic differences between pulmonary and thymic TC and AC [19, 32], there are currently no immunohistochemical markers that allow distinction between TNET and PNET in the absence of clinical and imaging data (Table 1). Of note, most thymic and pulmonary carcinoids are negative for TTF1 [18, 26, 33]. There are however a few interesting differences in the epidemiology of TNET and PNET: thymic TC and AC show a strong male predominance, while pulmonary carcinoids occur more often in females. AC and LCNEC are by far the most frequent subtypes in the thymus, while SCC and TC prevail in the lung. Most patients with pulmonary LCNEC and SCC are heavy smokers, while there is no established role of smoking in the development of any NET type in the thymus. There are also important epidemiological differences among the different TNET subtypes: as mentioned above, there is a striking male predominance (males are affected 3 to 6 times more frequently) for TC and AC, while LCNEC and SCC affect males and females equally. MEN1 is a risk factor only for TC and AC, while LCNEC and SCC are not observed in this setting. These observations are important because they point to substantial differences between low-grade (TC and AC) and high-grade (LCNEC and SCC) TNET. Most patients present with local symptoms (chest pain, cough, dyspnea, or superior vena cava syndrome) [11, 22, 30]. The vast majority of patients with paraneoplastic syndromes due to ectopic hormone production have TC or AC. These include Cushing syndrome (17–30%) [7, 30, 34], hypercalcemia/hypophosphatemia [42], or hyperparathyroidism [35]. In stark contrast to thymomas, TNET have a high propensity for regional lymph node metastases and > 50% of patients show involvement of regional lymph nodes at diagnosis [38]. The histological subtype is prognostically relevant: 5-year survival rates decrease from 50–70% in TC and AC [8, 22, 30, 32] to 30–66% in LCNEC [3–5, 9, 20, 24, 25, 29, 32, 36] and to 0% (median survival 13–26 months) in SCC [17, 23, 32, 36, 39]. The significant variation of published survival data in LCNEC is remarkable and points to a marked heterogeneity of the tumors analyzed.

**Table 1:** Immunohistochemical profiles of TNET (*n* = 45)*

% positive cases	CGA	EZH2	TTF1	Pax8	CD5	CD117	Calcitonin
TC (*n* = 10)	*100*	*0*	0	60	10	10	11
AC (*n* = 24)	*100*	*4*	0	42	8	13	4
LCNEC (*n* = 8)	*50*	*50*	25	63	38	25	0
SCC (*n* = 3)	*0*	*100*	0	0	0	0	0

*TC* typical carcinoids, *AC* atypical carcinoids, *LCNEC* large cell neuroendocrine carcinomas, *SCC* small cell carcinomas, *CGA* chromogranin A; TTF1 clone: 8G7G3/1; *unpublished own results and [37, 43]

### Molecular findings in TNET

**Fig. 1:**
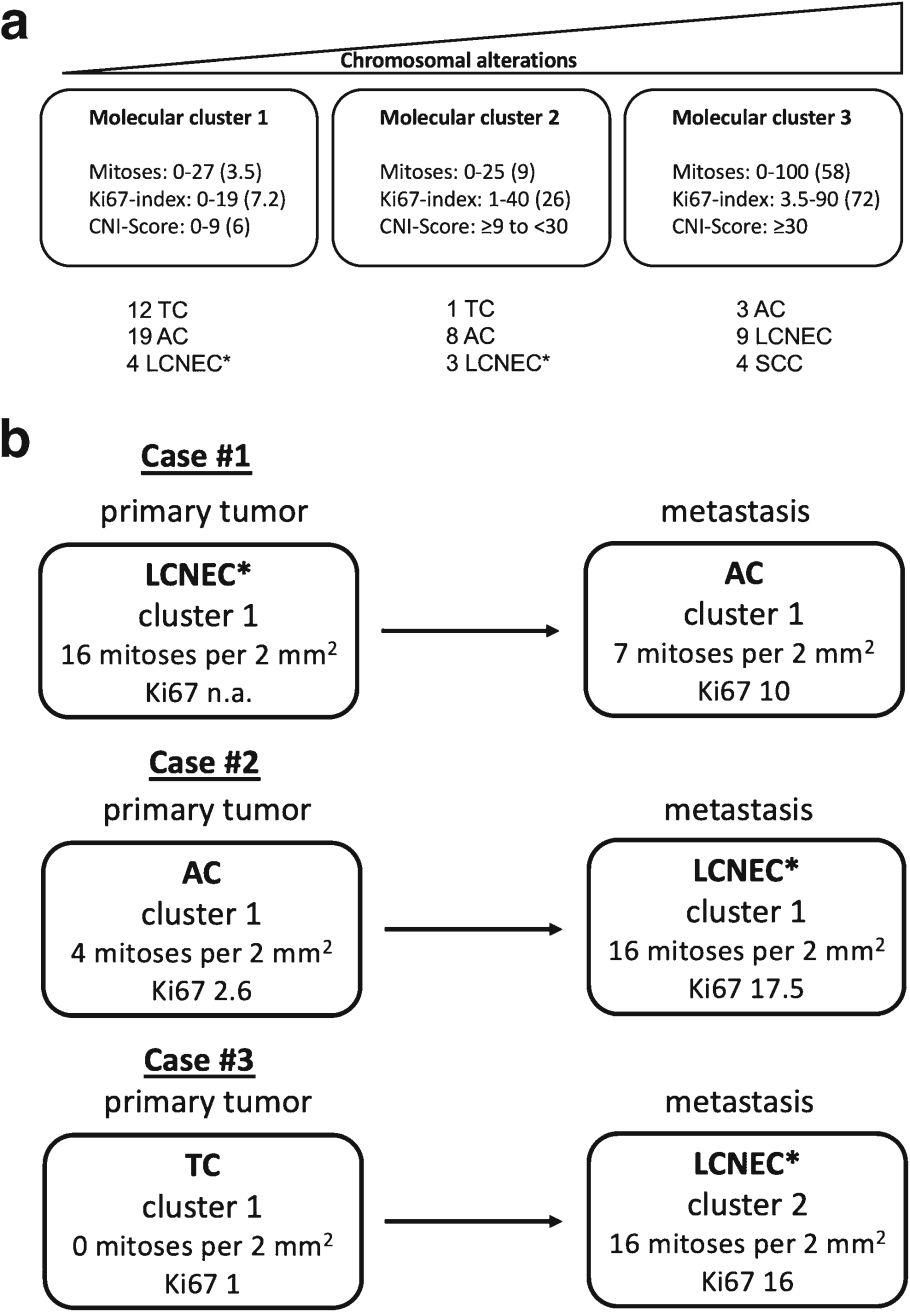
Molecular findings with impact on the conceptual classification of thymic neuroendocrine tumors (TNET). (**a**) Low-coverage whole-genome sequencing revealed three major molecular clusters with few (cluster 1), moderate (cluster 2), and high (cluster 3) numbers of chromosomal alterations. The numbers of chromosomal alterations were quantified using a chromosomal instability index (CNI-score). The graph depicts the distribution of histological subtypes among the three clusters (*TC* typical carcinoid, *AC* atypical carcinoid, *LCNEC* large cell neuroendocrine carcinoma)—cases marked with * were later re-classified as neuroendocrine tumors G3 (NET G3). *SCC* small cell carcinoma. (**b**) Comparison of three individual cases where more than one material was available

There are few published data on genomic features of TNET [8, 21, 27, 32] and virtually no data on the mutational spectrum of these tumors. One of the largest studies to date [32] using comparative whole-genome hybridization (CGH) found an incremental increase of genomic alterations from TC to AC and LCNEC/SCC that correlated with survival. Moreover, this study reported gene amplifications of *MYC* in LCNEC. A follow-up study using low-coverage (“shallow”) whole-genome sequencing confirmed this observation but found also a significant overlap between the profiles of AC and LCNEC [8]. The primary objective of this study was to use genomic data as “ground truth” in comparison to the histological classification according to current world health classification (WHO) criteria (Table 2). To compare individual cases and classes, the mapped reads were counted in windows (“bins”) along the chromosomes. The percentages of bins above/below the thresholds were calculated as a general measure of the amount of copy number aberrations present in each tumor (chromosomal instability score, CNI). Using this approach, the study identified three major molecular clusters with low (cluster 1), intermediate (cluster 2), and high CNI scores (cluster 3), for which cutoff values were statistically determined. Somewhat unexpectedly, this approach revealed significant “cluster infidelity” among the morphologic TNET subtypes: cluster 1 with few genomic alterations and low CNI score contained most TC and AC but also 4 LCNEC. In contrast, cluster 3 with most genomic alterations and highest CNI contained most LCNEC and all SCC but also 3 AC (Fig.1a). In addition, the authors found two extreme outliers: one atypical carcinoid with a very high CNI and one case classified as LCNEC according to WHO criteria (16 mitoses per 2 mm^2^) with very low CNI. Another highly informative observation came from a few cases where materials from the primary tumor and syn- or metachronous metastases were available (Fig. 1b). These cases showed heterogeneity between primary tumors and their metastases: patients had primary tumors classified, e.g., as typical carcinoid and metastases classified as LCNEC. A comparison of the genetic features of these cases showed mostly overlapping features with some additional alterations in the more progressed lesions. Remarkably, all of these cases belonged to the molecular clusters 1 and 2 with few and moderate numbers of chromosomal changes. Together, these observations have important implications: (1) The correlation between morphology and genetic complexity in TNET is imperfect—cases with “low-grade” morphology can have complex genetic features and vice versa. The same study showed that the molecular classification had at least the same prognostic relevance as current histologic classifications including WHO. (2) Cases that fall into the molecular clusters with low and intermediate complexity (cluster 1 and 2) form a spectrum, where morphological and molecular progression can occur. This spectrum includes cases that were classified as LCNEC according to current WHO criteria (these cases were provisionally termed NET G3 for better distinction from bona fide LCNEC within the highly complex cluster 3). (3) Progression of tumors from the molecular clusters 1 and 2 to the high-grade cluster 3 was not observed in this study and remains to be shown (in analogy to NET in other organs), suggesting at least two alternative molecular routes: the “low/intermediate” route that can lead to TC, AC, and NET G3 and the “high-grade” route that leads to SCC and LCNEC.

**Table 2:** WHO classification of neuroendocrine tumors of the thymus [31]

Current WHO classification		Low-grade	Intermediate-grade	High-grade	
	Morphological classification	Typical carcinoid	Atypical carcinoid	Large cell neuroendocrine carcinoma (LCNEC)	Small cell carcinoma
		No necrosis <2 mitoses per 2 mm^2^ (mean: 1 per 2 mm^2^)	Necrosis present (any) and/or·2–10 mitoses per 2 mm^2^ (mean: 6.5 per 2 mm^2^)	Non-small cell cytology Neuroendocrine markers > 10 mitoses per 2 mm^2^ (mean: 45 per 2 mm^2^) Frequent necrosis	Small cell cytology > 10 mitoses per 2 mm^2^ (mean: 110 per 2 mm^2^)

### Cases currently classified as LCNEC according to WHO criteria contain a group of tumors overlapping with carcinoids (“NET G3”)

**Fig. 2:**
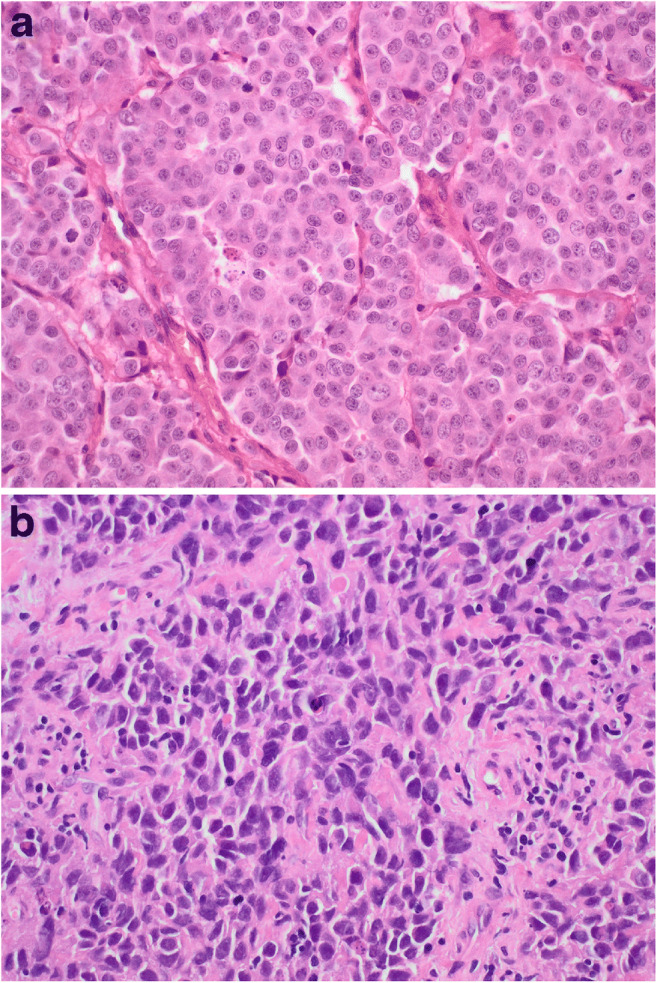
Representative histological images of neuroendocrine tumors G3 (NET G3) (**a**) and large cell neuroendocrine carcinomas (LCNEC) (**b**)

**Fig. 3:**
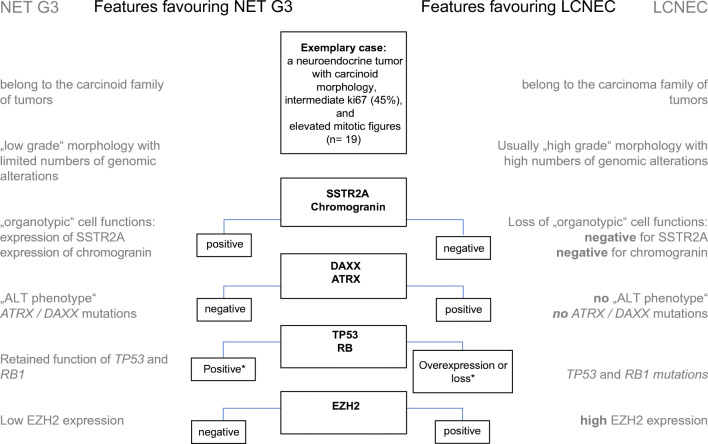
Immunohistochemical findings aiding in the distinction between neuroendocrine tumors G3 (NET G3) and large cell neuroendocrine carcinomas (LCNEC). *Immunohistochemical TP53 stainings suggestive of *TP53* gene mutations include overexpression and complete negative staining of tumor cell nuclei

In consequence of the conclusions described above, the authors next compared the NET G3 cases from the molecular clusters 1 and 2 to the “bona fide” LCNEC from the high-grade cluster 3 using a panel of immunohistochemical antibodies initially proposed by Yachida et al. [41] for the subtyping of pancreatic NET together with limited next-generation sequencing of selected genes. The two groups showed substantial differences. NET G3 invariably had carcinoid morphology (trabecular growth patterns, delicate vasculature, pepper-and-salt chromatin), while most LCNEC showed cytologic high-grade features (Fig. 2). Although LCNEC showed much higher ki67 and mitotic indices on average, these features were not helpful for the distinction of individual cases due to considerable overlap between the two groups. The best immunohistochemical markers for the distinction of NET G3 and LCNEC were chromogranin and EZH2: chromogranin was positive in all NET G3 but was lost in 4 out of 5 LCNEC (Fig. 3). Vice versa, EZH2 was negative in NET G3 and positive in LCNEC. Patients with EZH2-positive tumors showed a significantly shorter overall survival than patients with EZH2-negative tumors. EZH2 is a methyltransferase and is the functional component of the polycomb repressive complex 2 and a potent negative regulator of gene expression [6]. Overexpression of EZH2 is associated with poor survival, increased proliferation, and overexpression of TP53 [10] in many cancers including aggressive lung and gastrointestinal NET [2, 10]. Next-generation gene panel sequencing showed a single mutation of the gene encoding for alpha-thalassemia/mental retardation, X-linked (*ATRX*) in a NET G3. ATRX is a transcriptional regulator that belongs to the SWI/SNF family of chromatin remodeling proteins. ATRX and death-domain-associated protein (DAXX) interact with one another and are required for deposition of histone H3.3 at telomeres and other genomic repeats [40]. There is a strong correlation between ATRX and DAXX mutations and an alternative lengthening of telomeres (ALT) phenotype in pancreatic NET [12, 14]. Immunohistochemistry is a sensitive and specific screening tool for *ATRX* and *DAXX* mutations [12–14]. Although the loss of ATRX/DAXX and ALT in pancreatic NET is generally associated with tumor aggressiveness and reduced progression-free survival, these features are associated with better overall survival in the sub-cohort of metastatic patients [15]. In addition, the analysis revealed an unexpectedly high frequency of neurofibromin gene (*NF1*) mutations in 100% of NET G3 and LCNEC. NF1 inhibits RAS/MAPK signaling and is mutated in many cancers including soft tissue sarcomas, desmoplastic melanomas, and lung cancers [16].

## Conclusions and futures perspectives

Together, these findings lead to important conceptual changes in the classification of TNET (Table 3). Although the mitotic index is an important tool that helps to stratify patients and to predict prognosis [32], the current mitotic thresholds used to classify TNET in the WHO classification (maintained also in the upcoming version) are insufficient to cover the whole spectrum of tumors. Molecular findings indicate that TC, AC, and the recently discovered NET G3 form a continuum where morphologic and molecular progression can occur, e.g., during relapses or in metastases. These tumors share the expression of chromogranin and somatostatin receptor 2A (SSTR2A) in the absence of significant EZH2 expression. The stainings for TP53 and RB are unremarkable (“wild type”). Some tumors harbor mutations of *ATRX* (and presumably *DAXX*). Mitotic counts and ki67 index are usually much higher in true LCNEC and SCC, which often show loss of chromogranin and SSTR2A staining and overexpression of EZH2, accompanied by overexpression or complete loss of TP53 and/or RB1. Given the many similarities of TNET with NET in other organs, it is likely that the distinction between NET G3 and LCNEC will have therapeutic relevance: clinical experience has shown that gastrointestinal NET G3 show only limited response to platinum-based chemotherapy regimens used to treat patients with neuroendocrine carcinomas [28]. Recent molecular studies have identified a subgroup of tumors very likely corresponding to TNET G3 also in the lung [1]. It is currently unknown whether thymic NET G3 can progress to LCNEC or even SCC. The observation that NET G3 and LCNEC shared *NF1* gene mutations and the significantly overlapping genomic profiles of AC and LCNEC rather seem to indicate that this may be possible. It is to be hoped that the current concept will aid in clinical decisions and the design of scientific or clinical studies. Further work will be necessary to better characterize the mutational and gene expression or proteomic profile of thymic neuroendocrine tumors in comparison to the much better studied pulmonary NET.

**Table 3:** Evolving concept for the classification of thymic neuroendocrine tumors based on molecular data

Evolving concept		Low- and intermediate-grade NETs (TC, AC, NET G3)	High-grade NET (LCNEC, SCC)
	Immunohistochemical and molecular features	Tumors showing characteristic morphological and immunohistochemical neuroendocrine features NET G3 shows increased mitotic counts (11–27 per 2 mm^2^, mean 16.8) and ki67 index (15–66%, mean 30%) Low to intermediate numbers of chromosomal alterations *ATRX* gene mutations Somatostatin receptor (SSTR2A) positive Chromogranin positive EZH2 negative	High-grade morphology, often with loss of one or more immunohistochemical neuroendocrine markers High mitotic counts (12–100 per 2 mm^2^, mean 43.4) and high ki67 index (52–90%, mean 66%) High numbers of chromosomal alterations No *ATRX* gene mutations Somatostatin receptor (SSTR2A) negative Chromogranin mostly negative EZH2 mostly positive

*TC* typical carcinoids, *AC* atypical carcinoids, *LCNEC* large cell neuroendocrine carcinomas, *SCC* small cell carcinomas

## Declarations

This review does not contain primary data and complies with Ethical Standards and the Declaration of Helsinki.
